# Effects of repetitive transcranial magnetic stimulation on lower extremity motor function and optimal parameters in stroke patients with different stages of stroke: a systematic evaluation and meta-analysis

**DOI:** 10.3389/fneur.2024.1372159

**Published:** 2024-07-26

**Authors:** Shiyu Fan, Long Yan, Junfeng Zhang, Yulin Qian, Meng Wang, Lingqing Yang, Tao Yu

**Affiliations:** ^1^Comprehensive Rehabilitation Unit, The First Affiliated Hospital of Tianjin University of Traditional Chinese Medicine/National Clinical Research Center of Acupuncture and Moxibustion, Tianjin, China; ^2^Graduate School, Tianjin University of Traditional Chinese Medicine, Tianjin, China

**Keywords:** repetitive transcranial magnetic stimulation, stroke, lower extremity, balance, walking speed

## Abstract

**Background:**

Repetitive transcranial magnetic stimulation (rTMS), as an emerging non-invasive neuromodulation technique, is now widely employed in rehabilitation therapy. The purpose of this paper is to comprehensively summarize existing evidence regarding rTMS intervention for lower limb motor function in patients at different stages of stroke.

**Methods:**

A systematic search was conducted to identify randomized controlled trials (RCTs) assessing the efficacy of rTMS for treating lower limb motor dysfunction after stroke. Multiple databases, including China National Knowledge Infrastructure (CNKI), Wanfang Data Knowledge Service Platform, VIP Database, PubMed, Embase, Web of Science, and Cochrane Library, were searched. The search period extended from the inception of the libraries to June 2024. Literature information was extracted, and methodological quality was evaluated using the risk of bias assessment tool in the Cochrane Handbook. Meta-analysis was performed using Stata 17.0 software.

**Results:**

Overall, 49 appropriate studies (including 3,558 stroke subjects) were found. Meta-analysis results demonstrated that rTMS effectively improved lower limb motor function across all stages of stroke. The intervention was particularly more effective in patients in the subacute stage than in the acute or chronic stages. Subgroup analysis revealed that, for acute-stage patients, low-frequency stimulation targeting the M1 or DLPFC brain regions on the unaffected side with 20–40 sessions significantly improved FMA-LE scores. In subacute-phase patients, low-frequency stimulation targeting the M1 brain regions on the unaffected side with 18 sessions significantly improved FMA-LE scores. The results demonstrated that HF-rTMS was more effective than LF-rTMS in improving walking speed, with the greatest efficacy observed at 20 sessions. While for enhancing gait balance in stroke patients, LF-rTMS with the best therapeutic effect was observed at a frequency of 20–40 treatments.

**Conclusion:**

This study demonstrates the efficacy of rTMS in improving lower limb motor function, balance, and walking speed in stroke patients at various stages. The findings provide a valuable reference for the development of optimized rTMS treatment plans in clinical practice.

**Systematic review registration**: PROSPERO: CRD42023466094.

## 1 Introduction

Stroke, ranked as the second leading cause of global mortality and disability, exhibits escalating incidence, disability, and mortality rates annually, imposing a substantial societal burden. 70–80% of stroke patients suffer from varying degrees of limb dysfunction, profoundly affecting daily activities and diminishing quality of life (1, 2). Given the pivotal role of walking in daily life, particularly in averting risk factors associated with reduced mobility or prolonged bed rest, the rehabilitation goals for stroke patients now emphasize the imperative of improving lower limb function and restoring gait (3).

Diverse treatments for post-stroke lower limb dyskinesia have emerged, encompassing rehabilitation training, acupuncture, and neuromodulation techniques. However, traditional rehabilitation training proves time-consuming and necessitates a specific limb function level. Acupuncture’s mechanism remains elusive, lacking a standardized treatment protocol. Presently, non-invasive neuromodulation techniques, notably Repetitive Transcranial Magnetic Stimulation (rTMS), have garnered favor in stroke rehabilitation due to their non-invasiveness, painlessness, and operational simplicity.

rTMS emerges as a non-invasive technique utilizing electromagnetic induction to depolarize superficial axons, thereby altering the excitatory state of neurons and activating cortical networks (4, 5). This technique has gained widespread use in the rehabilitation treatment of stroke patients due to its non-invasive, painless, and straightforward operation. The efficient promotion of limb function recovery through various mechanisms, including the regulation of cortical excitability, alteration of neurological plasticity, modulation of brain network function, improvement of cerebral glucose metabolism, and regulation of microglial cell polarization, underscores the multifaceted benefits of rTMS (6–10).

As neuromodulation technology evolves, a novel form of rTMS therapy has emerged. Altering the shape, stimulation mode, and intensity of the stimulation coil allows for the activation of deeper brain tissues (11). This innovation has been demonstrated by Liao et al. (12), showcasing the efficiency of intermittent theta burst stimulation (iTBS) targeting the contralateral cerebellum in rapidly improving balance and motor functions in post-stroke patients. Additionally, Dionísio (13) found that continuous theta-burst stimulation (cTBS) significantly improved neurophysiological effects in subacute stroke patients and positively contributed to motor function recovery in post-stroke patients.

While previous studies have predominantly focused on exploring the efficacy of rTMS on motor function in stroke patients (14, 15), there is a relative paucity of studies on the effects of rTMS on improving lower limb motor function and gait at different stages of the disease. Therefore, this systematic review aims to investigate the effect of TMS intervention on functional recovery of the lower limbs in patients with different stages of stroke (acute [<1 month], subacute [1–6 months] and chronic [>6 months]) (16, 17). Besides, this study focuses on analyzing the differences in the effects of various stimulation modes, stimulation frequency, stimulation brain region, stimulation hemisphere, and the treatment course of rTMS on the lower limb function and gait parameters of stroke patients. The objective is to summarize the optimal stimulation parameters for rTMS intervention in lower limb dysfunction and gait abnormality of stroke patients, providing a scientific basis and data support for the development of clinical rehabilitation programs using rTMS in the post-stroke period.

## 2 Methods

### 2.1 Protocol and search strategy

This study adhered to the PRISMA 2020 statement and Cochrane Review’s Handbook 5.1 guidelines. Furthermore, it was prospectively registered with PROSPERO under the identifier CRD42023466094. Since the study involved the synthesis of data from previously published studies, ethical review board approval was not required.

A comprehensive search of both Chinese and English databases was executed to retrieve clinical research literature related to lower limb movement and gait in stroke patients treated with rTMS, spanning from the inception of the databases to June 2024. Chinese databases, including China National Knowledge Infrastructure (CNKI), Wanfang Data Knowledge Service Platform, and VIP Database, were surveyed. English databases, including Embase, PubMed, Web of Science, and Cochrane Library, were systematically searched. Search terms included keywords associated with stroke, motor function, TMS and related terms (search strategy of PubMed is presented in Supplementary Table S1). No restrictions were placed on ethnicity, the language of publication, or the type of journal published.

### 2.2 Inclusion criteria

1. Study type: A randomized controlled trial.
2. Type of language: English, Chinese.
3. Subjects: This trial included adult patients (age ≥ 18 years) diagnosed with stroke based on relevant clinical examinations such as CT, MRI, etc. The patients had residual lower limb motor dysfunction (FMA-LE values<34) or gait abnormality (examples include decreased walking speed and balance dysfunction) after stroke, clear consciousness, and were cooperative with treatment.
4. Interventions: The intervention group received TMS alone or TMS combined with additional interventions, while the control group received sham TMS (STMS) or no TMS.
5. Outcome Indicators: The Lower Extremity portion of the Fugl-Meyer Rating Scale (FMA-LE), tests of walking speed (such as the 10-meter walking speed test [10MWS] and quantitative stride analysis), and measures of balance function (such as the balance subscales of any scale, including the Berg Balance Scale [BBS]) are used for assessment.
6. For duplicate publications, the latest published edition was included.

### 2.3 Exclusion criteria

1. Comorbidity with psychiatric or other malignant disease or contraindication to receiving TMS (e.g., pacemaker, metal objects in the head, or history of epilepsy).
2. Uncontrolled single-arm trials, animal experiments, case reports, systematic evaluations, reviews, expert experience, and conference papers.
3. No access to relevant data or full text.
4. Duration of intervention less than 2 weeks.

### 2.4 Data extraction

Based on Microsoft Excel processing software, entry was performed independently by two medical researchers (Fan Shiyu, Yang Lingqing), and entry elements mainly included basic literature information (authors, publication year, and sample size), basic clinical literature information (gender, age-structure data, and disease duration), the interventions (treatment interventions including the type of rTMS, stimulation frequency, number of impulses, stimulation site, and treatment schedule)， and mean difference (MD) and standard deviation (SD) of the main outcome indicators (including FMA-LE score, balance test, walking speed test). Records were cross-checked and disagreements were adjudicated by a third independent investigator.

### 2.5 Quality assessment

The quality of the included literature was assessed according to the evaluation criteria of the Cochrane Handbook version 5.1.0 manual in the United States. The quality was categorized into 3 levels of high, medium, and low risk and was independently assessed and proofread by 2 researchers, with disputes adjudicated by a third independent researcher.

### 2.6 Data synthesis and analysis

This study investigates the effect of rTMS on lower limb motor function and gait after different stages of stroke. The included pilot studies were categorized into acute (<1 month), subacute (1–6 months), and chronic (>6 months) phases of stroke according to the average time of subjects since stroke (18). According to the guideline request (19), we finally chose to use the FMA-LE scale to assess lower limb motor function, the BBS and FMBS scales to assess gait balance disorders, and the 10 m MWS/6 m MWS/Gait analysis software, to assess walking speed.

For statistical analysis of the data, Stata17.0 software was used. In this study, the outcome indicators were continuous variables. When evaluating balance disorders and walking speed in stroke patients, the Standard Mean Difference (SMD) and its 95% CI were chosen to be used due to the existence of inconsistency in the way the same indicators were evaluated. In contrast, the assessment of lower limb function using the FMA-LE scale was consistent. Therefore, the Mean Difference (MD) and its 95% CI were used. The heterogeneity of the data should be assessed before combining the effect sizes of the outcome indicators. In this study, the Cochrane Q test and I^2^ test were used to assess the statistical heterogeneity of the included literature; if I^2^ ≤ 50%, it indicated that the statistical heterogeneity among the literature was low, and the fixed-effects model was used for meta-analysis; if I^2^ > 50%, it indicated that the statistical heterogeneity among the literature was high, and the random-effects model was used for the meta-analysis. Stata17.0 software was used to conduct sensitivity analysis and meta-regression analysis to explore the possible sources of heterogeneity in the literature and to propose hypotheses about the causes of heterogeneity. Publication bias analysis was performed by drawing funnel plots and combining them with Egger’s test to further clarify whether there was publication bias or a small sample effect in the included studies.

## 3 Results

### 3.1 Research search

**Figure 1:**
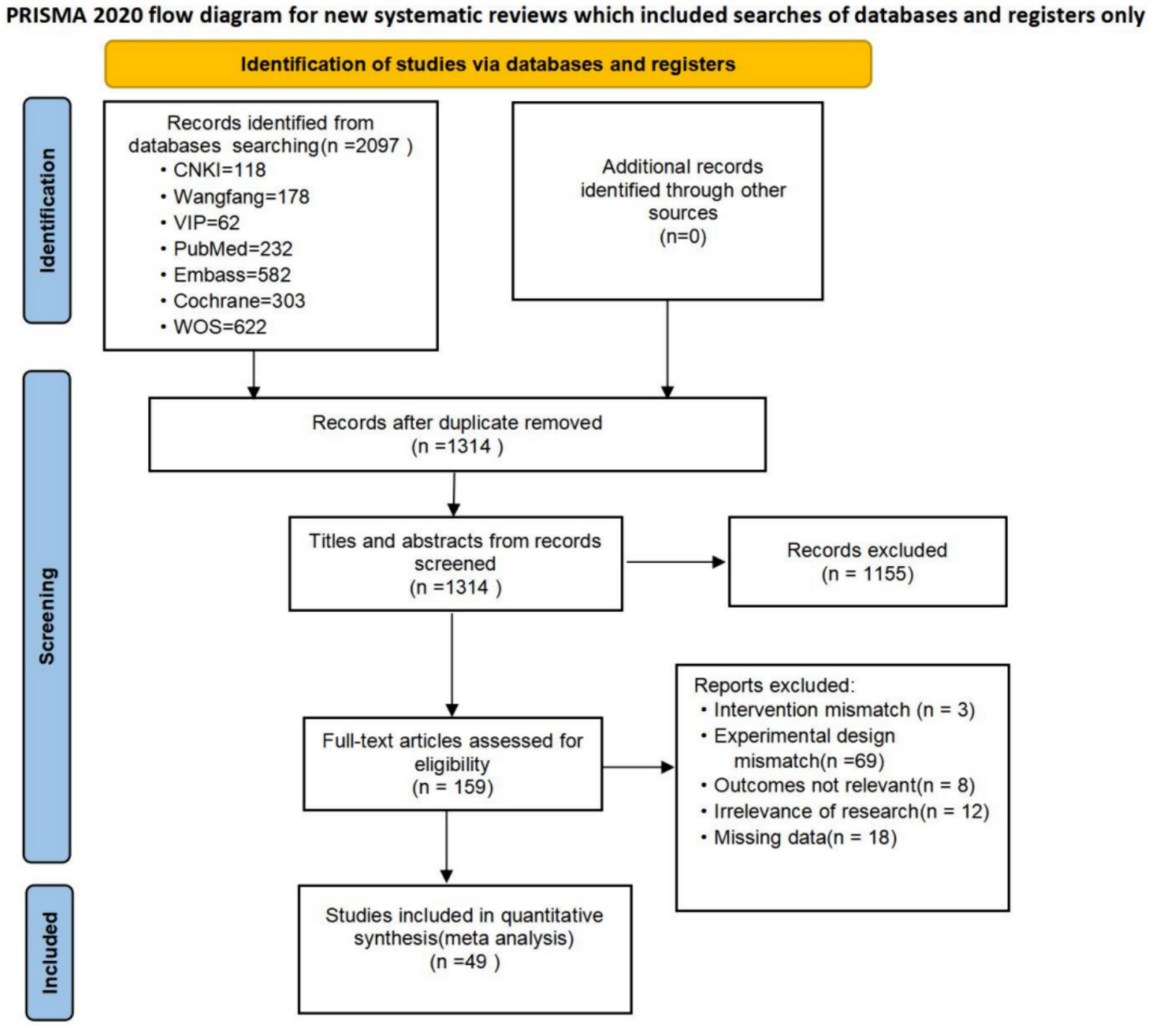
PRISMA flow chart on selection and inclusion of studies.

A total of 2097 articles were searched. 783 duplicates were removed using NoteExpress (Tianjin University of Traditional Chinese Medicine Library Edition). After reading the abstracts, 1,155 articles were excluded. and after reviewing the full text, 110 articles were excluded, of which 3 article did not match the interventions, 69 articles did not match the experimental design, 8 articles did not match the outcome measures, 12 articles did not match the study content, and 18 articles did not have complete data. Finally, we included 49 randomized controlled studies with a total of subjects. The process of screening the literature is detailed in Figure 1.

### 3.2 Study characteristics

A total of 49 studies were included in this analysis. 38 were in Chinese and 11 were in English. Primary outcome indicators included FMA-LE values in 39 studies; balance scales in 29 studies; and walking pace in 17 studies. The studies enrolled a total of patients, with 1816 in the control group and 1840 in the treatment group. The treatment group received rTMS therapy, while the control group underwent rehabilitation training. The basic characteristics of the literature are shown in Supplementary Table S2.

### 3.3 Quality evaluation

**Figure 2:**
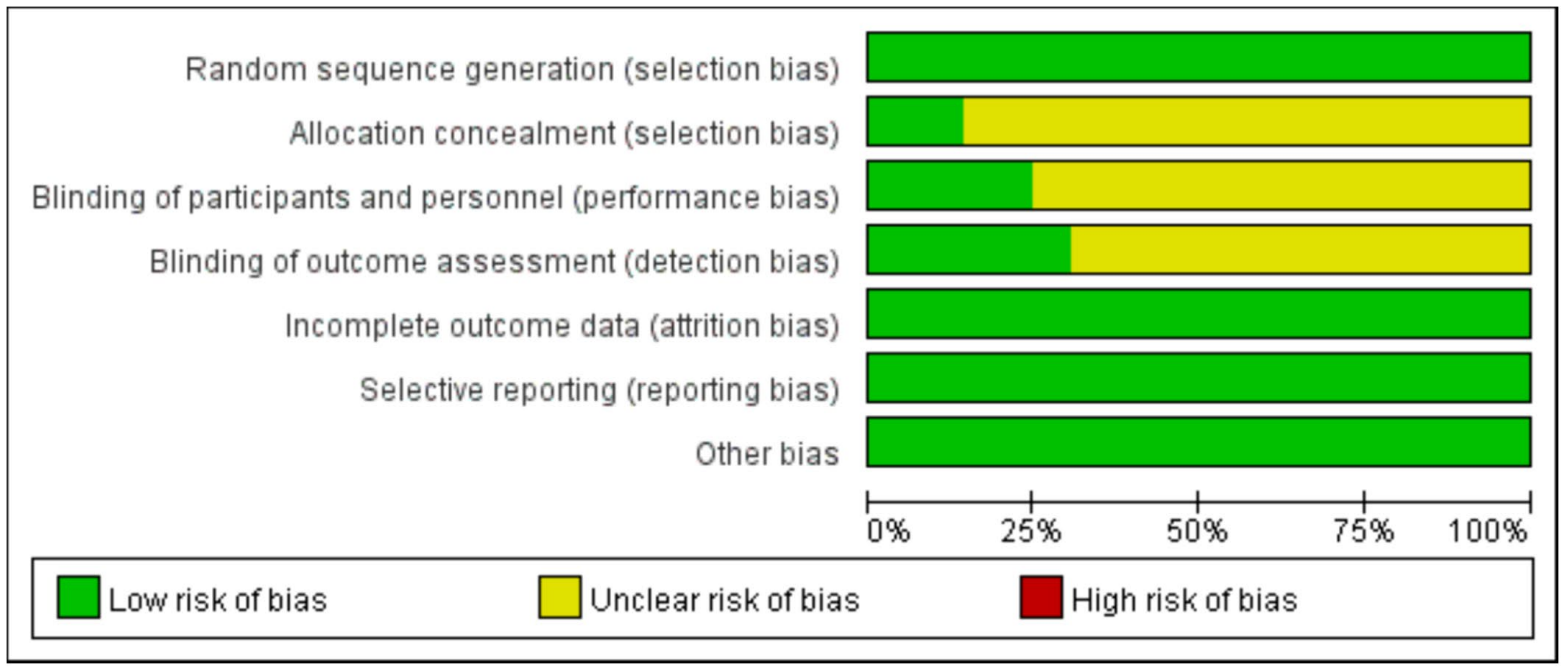
Graph of risk of bias for evaluation of included studies in this system.

**Figure 3:**
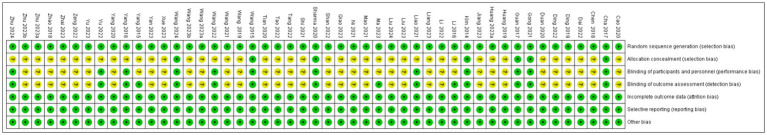
Summary of the risk of bias in this systematic evaluation of included studies.

All 49 included studies employed random allocation, with 39 studies (12, 20–57) using “random number table” grouping and 10 studies (58–67) using “lottery” grouping. 12 studies mention the implementation of participants and personnel blindness. 15 studies refer to the implementation of outcome assessment blindness. 7 studies reported on the design and implementation of allocation concealment. Furthermore, none of the 49 studies had attrition, deaths, or apparent selective reporting, and other sources of bias, leading to a low risk of bias. The detailed results of the risk of bias analysis are presented in Figures 2, 3.

### 3.4 Meta-analysis results

#### 3.4.1 Effect of rTMS on lower limb motor function in patients with different stages of stroke

**Figure 4:**
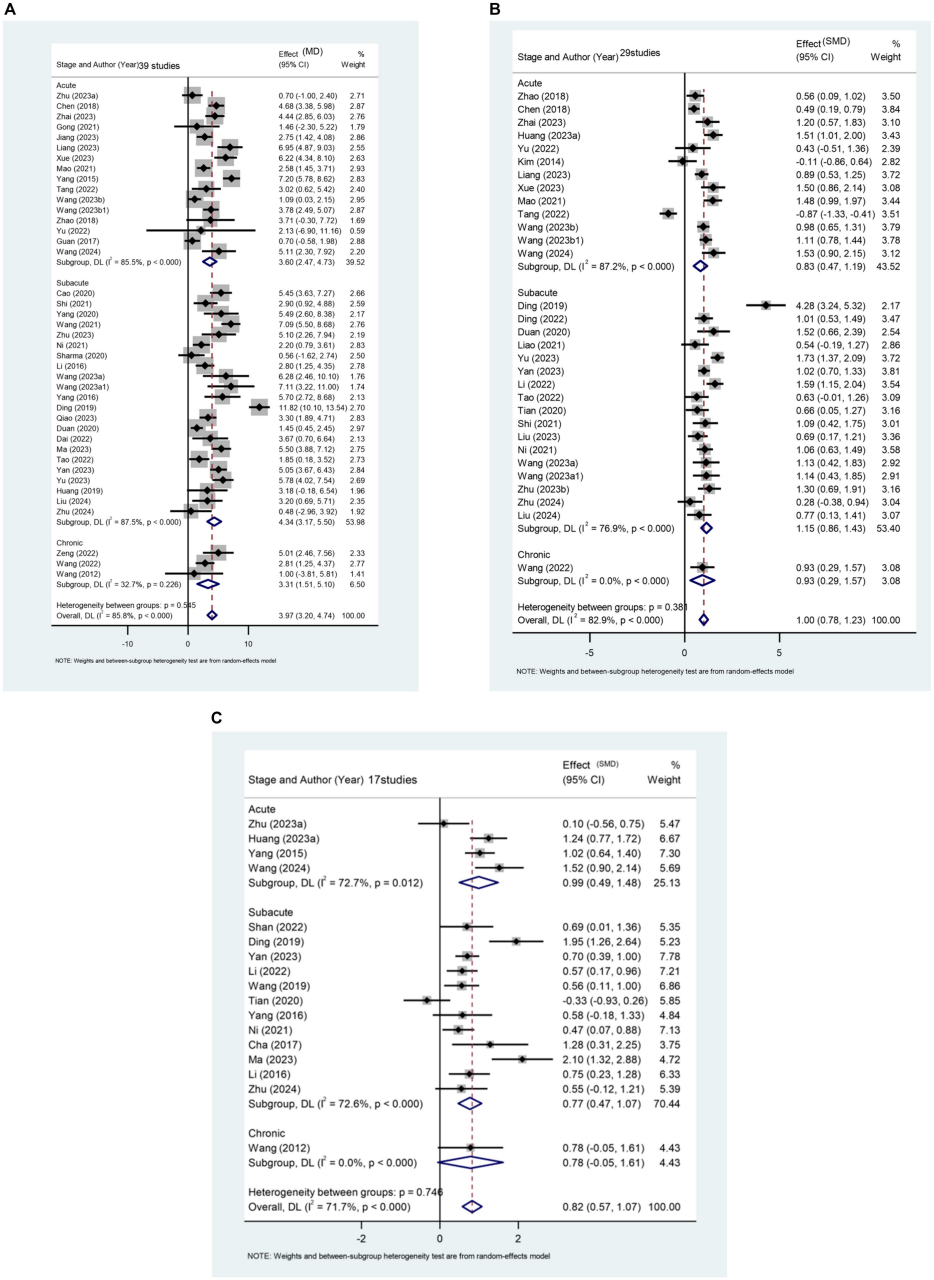
**(A)** Forest plot of FMA-LE in patients stroke compared with controls. **(B)** Forest plot of balance function in stroke patients compared with controls. **(C)** Forest plot of walking pace in stroke patients compared with controls.

This study assesses the impact of rTMS on the lower limb motor function of stroke patients across different stages, considering FMA-LE score, balance function, and walking speed. The results reveal a significant difference between the rTMS group and the sham stimulation group [MD (95% CI): 3.968 (3.199, 4.737), *p* < 0.001] in enhancing the FMA-LE of stroke patients (Figure 4A). Furthermore, rTMS proves effective in improving balance function (Figure 4B) and increasing walking speed (Figure 4C).

A total of 39 papers involving 3,013 patients report the effect of rTMS on FMA-LE scores in stroke patients. Subgroup analysis results (Figure 4A) indicate that rTMS is more effective in patients with subacute stroke [MD (95% CI): 4.336 (3.170, 5.501), *p* < 0.001]. This finding also applies to rTMS intervention for balance function in stroke patients (Figure 4B). The recovery of balance function was significantly better in patients in the subacute phase [SMD (95%CI): 1.147 (0.864, 1.429), *p* < 0.001] compared to those in the acute phase [SMD (95%CI): 0.829 (0.469, 1.188), *p* < 0.001] and patients in the chronic phase [SMD (95%CI): 0.932 (0.294, 1.571), *p* = 0.004].

Regarding the improvement of walking speed, subgroup analysis results (Figure 4C) indicate that rTMS had the most significant therapeutic effect on patients in the acute phase of stroke [SMD (95% CI): 0.899 (0.421, 1.377), *p* < 0.001].

#### 3.4.2 Optimal parameters for rTMS to improve FMA-LE in patients with acute phase stroke

**Figure 5:**
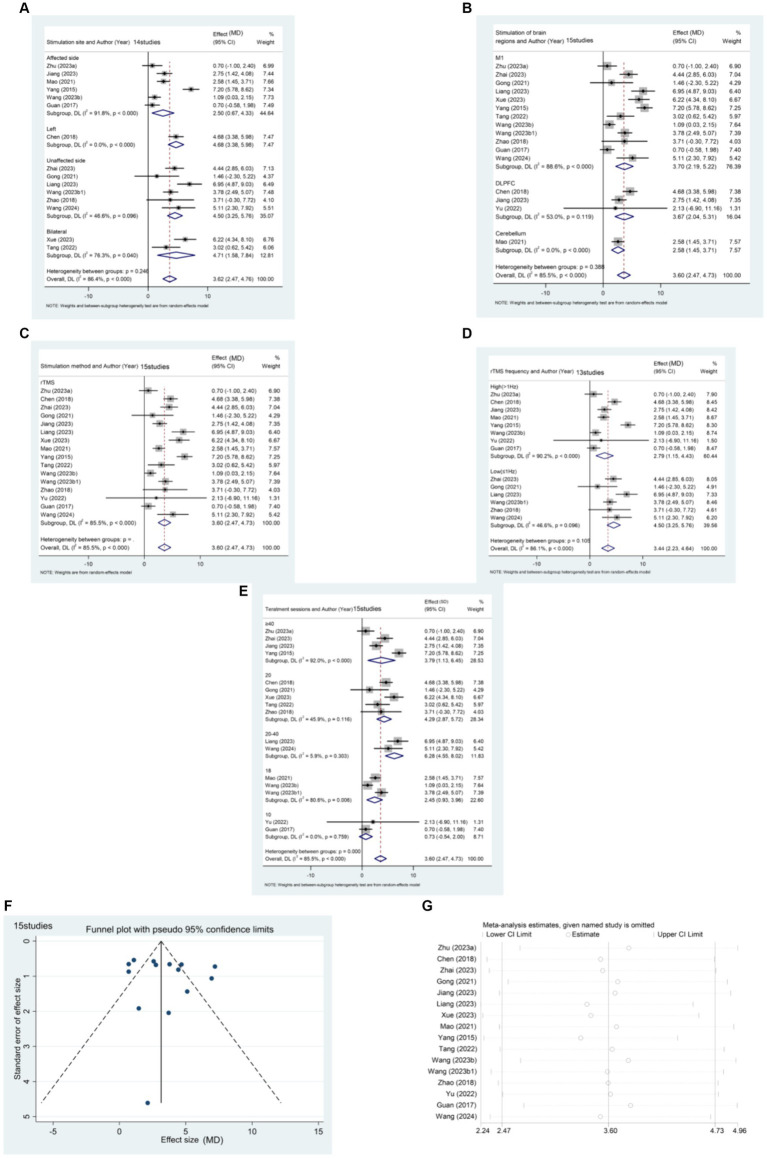
**(A)** Forest plot of FMA-LE in patients with acute phase stroke disaggregated by stimulate site compared with controls. **(B)** Forest plot of FMA-LE in patients with acute phase stroke disaggregated by stimulate of brain regions compared with controls. **(C)** Forest plot of FMA-LE in patients with acute phase stroke disaggregated by stimulate method compared with controls. **(D)** Forest plot of FMA-LE in patients with acute phase stroke disaggregated by stimulate frequence compared with controls. **(E)** Forest plot of FMA-LE in patients with acute phase stroke disaggregated by stimulate sessions compared with controls. **(F)** Funnel plot of FMA-LE in patients with acute phase stroke. **(G)** Results of sensitivity analysis in patients with acute phase stroke.

15 articles (21, 23, 24, 26, 32, 35, 38, 40, 46, 49, 52, 57, 59, 64, 67) reported the effect of rTMS in the acute post-stroke phase (≤1 month) in a total of 1,344 patients (Figure 5). The results of the subgroup analysis showed that rTMS intervention in the affected brain [MD (95% CI): 2.501 (0.667, 4.335), *p* = 0.008], unaffected brain [MD (95%CI): 4.503 (3.250, 5.755), *p* < 0.001], and bilateral [MD (95%CI):4.711 (1.580, 7.842), *p* = 0.003] were all statistically significant. Specifically, rTMS targeting M1 [MD (95% CI): 3.705 (2.193, 5.216), *p* < 0.001], DLPFC [MD (95% CI): 3.671 (2.037, 5.305), *p* < 0.001], and cerebellum [MD (95% CI): 2.580 (1.454, 3.706), *p* < 0.001] showed significant effects, respectively. The stimulation effect of targeting the M1 was slightly better than that of stimulating other brain regions. Patients in the acute phase were mainly treated with conventional rTMS [MD (95% CI): 3.597 (2.468, 4.725), *p* < 0.001]. Regarding the frequency of stimulation, the results indicate that both High-frequency rTMS [HF-rTMS; MD (95%CI): 2.794 (1.154, 4.434), *p* = 0.001] and Low-frequency rTMS [LF-rTMS; MD (95%CI):4.503 (3.250, 5.755), *p* < 0.001] treatments significantly improved the lower limb function of stroke patients in the acute phase. Regarding treatment duration, it appears that a higher number of treatments (>10) may result in better efficacy. Specifically, patients who underwent 20–40 treatments [MD (95% CI): 6.284 (4.550, 8.017), *p* < 0.001] experienced the most significant functional recovery in their lower extremities.

The funnel plot was generated using Stata 17.0 software, and Egger’s test was conducted to assess the presence of publication bias in the included literature. The funnel plot exhibited approximate symmetry between the left and right, with one study significantly biasing the funnel. Egger’s test resulted in a *p*-value of 0.427 (> 0.05), suggesting that studies with the outcome index of FMA-LE score have less publication bias for patients in the acute phase of stroke.

Sensitivity analysis was performed using Stata 17.0 software. The method of excluding each study one by one was applied, as illustrated in the figure below. The analysis revealed that none of the literature had a substantial impact on the study results. This indicates that the meta-analysis results for stroke duration ≤1 month and the outcome metric of the FMA-LE score were stable.

#### 3.4.3 Optimal parameters for rTMS to improve FMA-LE in patients with subacute phase stroke

**Figure 6:**
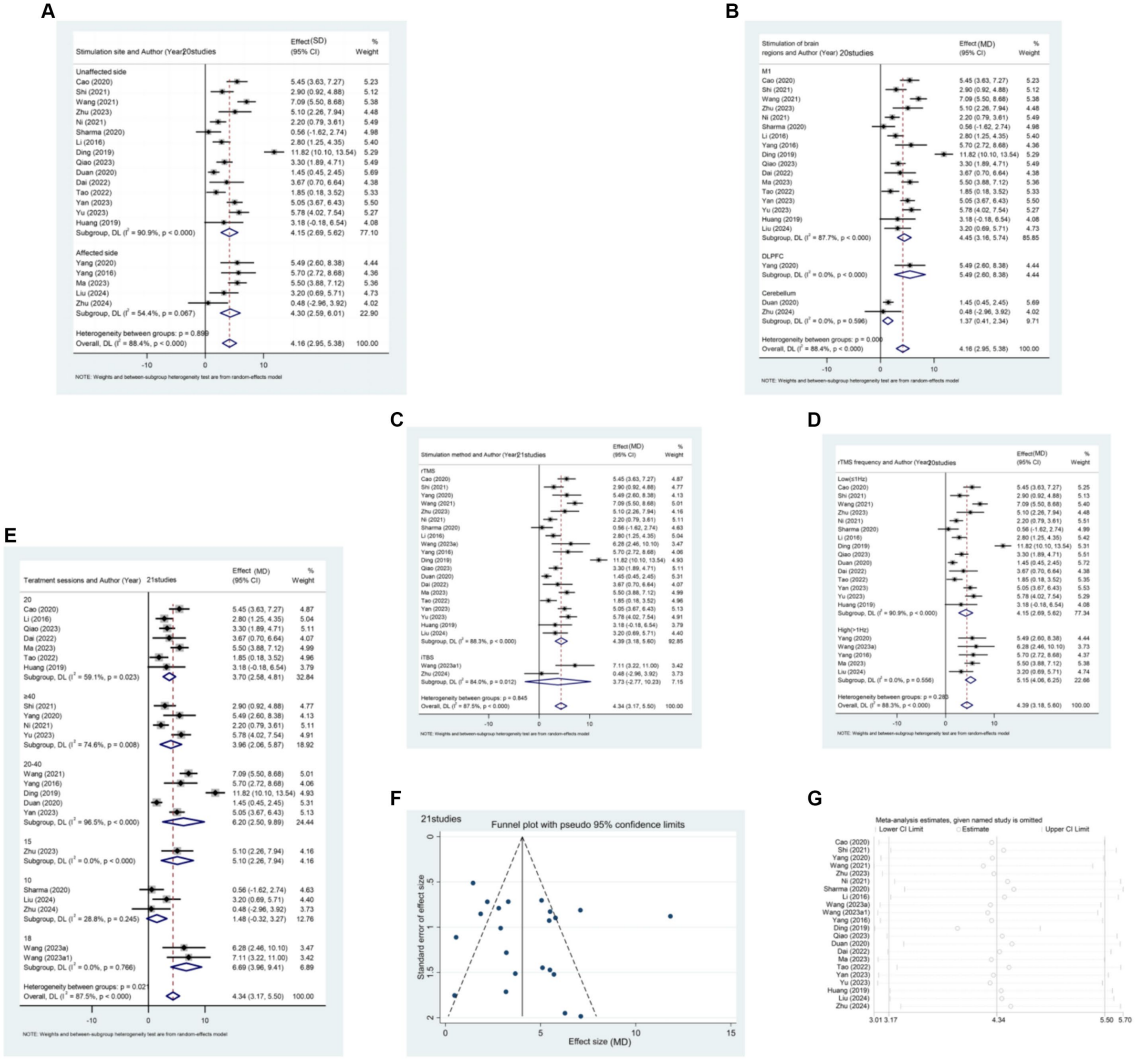
**(A)** Forest plot of FMA-LE in patients with subacute phase stroke disaggregated by stimulate site compared with controls. **(B)** Forest plot of FMA-LE in patients with subacute phase stroke disaggregated by stimulate of brain regions compared with controls. **(C)** Forest plot of FMA-LE in patients with subacute phase stroke disaggregated by stimulate method compared with controls. **(D)** Forest plot of FMA-LE in patients with subacute phase stroke disaggregated by stimulate frequence compared with controls. **(E)** Forest plot of FMA-LE in patients with subacute phase stroke disaggregated by stimulate sessions compared with controls. **(F)** Funnel plot of FMA-LE in patients with subacute phase stroke. **(G)** Results of sensitivity analysis in patients with subacute phase stroke.

21 articles (12, 22, 25, 29, 31, 33, 34, 37, 41, 42, 47, 48, 50, 51, 53–56, 61, 62, 66) reported the effect of rTMS on lower limb motor dysfunction in patients in the subacute phase (1–6 months) after stroke. The studies included a total of 1,505 patients, and the results were measured using the FMA-LE (Figure 6). The subgroup analysis results indicate that rTMS was significantly effective on both the affected hemisphere [MD (95% CI): 4.301 (2.589, 6.013), *p* < 0.001] and the unaffected hemisphere [MD (95% CI): 4.155 (2.692, 5.618), *p* < 0.001]. Additionally, the therapeutic efficacy of stimulating the affected hemisphere was superior to that of stimulating the unaffected side. Analyzing the stimulated brain regions, the stimulation of the M1 brain region [MD (95% CI): 4.445 (3.155, 5.735), *p* < 0.001] and DLPFC [MD (95% CI): 5.490 (2.604, 8.376), *p* < 0.001] was found to be superior to that of the cerebellum [MD (95% CI): 1.374 (0.410, 2.337), *p* = 0.005]. Our results also showed that conventional rTMS [MD (95%CI): 4.388 (3.181, 5.596), *p* < 0.001] was the most effective. The HF-rTMS treatment [MD (95% CI): 5.155 (4.063, 6.246), *p* < 0.001] was more effective than the LF-rTMS treatment [MD (95% CI): 4.155 (2.692, 5.618), *p* < 0.001]. The results of the stimulation sessions were similar to those of the acute phase. All rTMS stimulations greater than 10 showed significant differences. The stimulation session of 18 [MD (95% CI): 6.688 (3.961, 9.415), *p* < 0.001] and 20–40 [MD (95% CI): 6.199 (2.504, 9.894), *p* < 0.001] had the best treatment effect.

The funnel plot exhibited rough symmetry between the left and right, with one study significantly biasing the funnel. Egger’s test resulted in a *p*-value of 0.362 (> 0.05), suggesting that there was less publication bias for the outcome index of FMA-LE score in patients with subacute stroke.

The method of excluding each study one by one was employed for sensitivity analysis, and the results are depicted in the figure below. It was observed that none of the literature had a substantial impact on the study results. This indicates that the meta-analysis of patients with the subacute stage of stroke and the outcome indicator being FMA-LE score had high result stability.

#### 3.4.4 Effect of rTMS on FMA-LE in patients with chronic phase stroke

**Figure 7:**
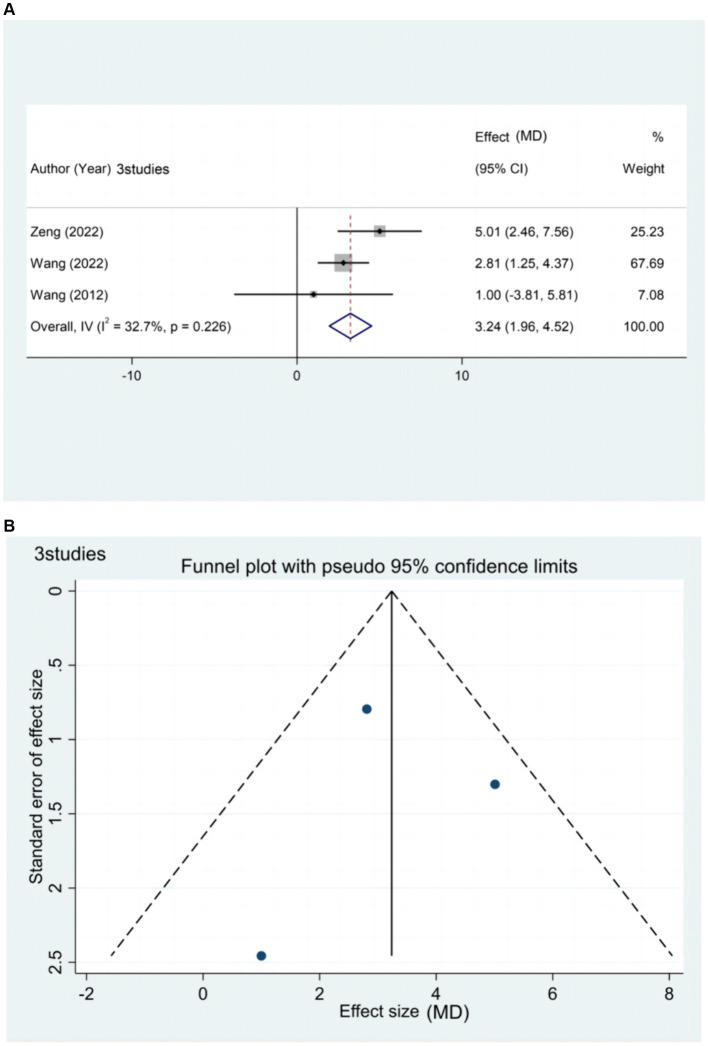
**(A)** Forest plot of FMA-LE in patients with chronic phase stroke. **(B)** Funnel plot of FMA-LE in patients with chronic phase stroke.

Three studies (20, 44, 65) reported the effect of rTMS treatment on FMA-LE in patients with chronic stroke (>6 months), including 146 patients (Figure 7). Subgroup analysis based on relevant stimulation parameters was not possible due to the limited amount of literature. However, the results of the meta-analysis indicate that rTMS can effectively improve lower limb motor dysfunction in patients with chronic stroke.

The funnel plot exhibited a symmetrical distribution between the left and right sides. Egger’s test resulted in a *p*-value of 0.954 (> 005), suggesting a small publication bias for the outcome index FMA-LE score in patients with chronic stages of stroke.

Sensitivity analysis was not performed in this study due to the limited number of included literature related to the chronic phase of stroke and the low heterogeneity among the literature (I2 = 32.7% ≤50%).

#### 3.4.5 Optimal parameters for rTMS to improve balance function in patients with stroke

29 articles (12, 21, 22, 24, 26–30, 32–34, 36, 38–44, 46, 49, 50, 52–54, 60, 66, 67) reported the effect of rTMS on lower limb motor dysfunction and balance scale scores in 2288 stroke patients (Supplementary Figure 1). According to the results of subgroup analysis, from the perspective of stimulating hemispheres, TMS is considered ineffective in bilateral hemispheric stimulation [SMD (95%CI): 0.31 (−2.01, 2.62), *p* = 0.797]. There was a marked difference in patients’ balance scale scores before and after treatment, with the most obvious efficacy observed in stimulation of the unaffected hemisphere [SMD (95%CI):1.241 (0.961, 1.521), *p* < 0.001]. Based on the analysis of stimulated brain regions, rTMS stimulation targeting the M1 brain region [SMD (95%CI): 1.131 (0.801, 1.462), *p* < 0.001] was found to have the most powerful therapeutic effect, followed by stimulation of the cerebellum [SMD (95%CI): 0.825 (0.397, 1.253), *p* < 0.001], and lastly by stimulation targeting the DLPFC brain region [SMD (95% CI): 0.530 (0.281, 0.778), *p* < 0.001]. In terms of stimulation pattern, traditional rTMS [SMD (95%CI):1.044 (0.800, 1.287), *p* < 0.001] was found to be more efficacious than iTBS [SMD (95%CI): 0.721 (0.341, 1.101), *p* < 0.001] for post-stroke balance deficits. After HF-rTMS [SMD (95%CI): 0.998 (0.786, 1.210), *p* < 0.001] and LF-rTMS [SMD (95%CI):1.191 (0.911, 1.471), *p* < 0.001] interventions, the patient’s balance disorders distinctly improved. LF-rTMS was found to be more effective. The patients were analyzed based on the number of stimulation sessions they received: between 20 and 40 sessions [SMD (95% CI): 1.421 (0.892, 1.950), *p* < 0.001], 40 sessions [SMD (95% CI): 1.314 (0.979, 1.649), *p* < 0.001], and over 40 sessions [SMD (95% CI): 1.406 (0.750, 2.062), *p* < 0.001]. The results indicate that a higher number of stimulation sessions had a more pronounced treatment effect.

The funnel plot exhibited approximate symmetry between the left and right sides, with two studies significantly biasing the funnel. However, Egger’s test (*p* = 0.526 > 0.05) indicates that publication bias was less prevalent in studies where the Balance Scale score was the outcome indicator.

The sensitivity analysis, conducted by excluding each study one by one, is illustrated in the figure below. It was observed that none of the literature had a substantial impact on the study results. This suggests that the results of the meta-analysis with the Balance Scale score as the outcome indicator were relatively robust.

#### 3.4.6 Optimal parameters for rTMS to improve walking speed in patients with stroke

17 articles (23, 27, 30, 33, 36, 37, 43, 45, 47, 50, 56–58, 63, 65–67) reported the effect of rTMS for lower limb motor dysfunction on walking speed after stroke in a total of 1,081 patients (Supplementary Figure 2). The results of the combined subgroup analysis showed that stimulation on the unaffected side of the brain [SMD (95% CI): 0.873 (0.613, 1.134), *p* < 0.001] was more influential in improving walking speed in stroke patients than stimulation on the affected side [SMD (95% CI): 0.728 (0.167, 1.288), *p* = 0.011]. And LF-rTMS [SMD (95% CI): 0.873 (0.613, 1.134), *p* < 0.001] was considered more effective than HF-rTMS [SMD (95% CI): 0.763 (0.103, 1.422), *p* = 0.023]. In terms of stimulation sessions, all stimulation protocols were statistically significant, and data analysis showed that the most significant improvement in patients’ gait speed was achieved with 20 stimulation sessions [SMD (95% CI): 1.073 (0.300, 1.846), *p* = 0.007].

The funnel plot exhibited approximate symmetry between the left and right, with three studies notably skewing the funnel. However, Egger’s test yielded a *p*-value of 0.89 (>0.05), indicating that studies with the outcome measure of walking speed exhibited less publication bias.

The sensitivity analysis of the included literature use the method of excluding each study one by one. As depicted in the figure below, it was observed that none of the literature had a substantial impact on the study results. This suggests that the meta-analysis results for the outcome measure of walking speed were stable.

## 4 Discussion

rTMS has gained widespread use in addressing post-stroke lower limb dyskinesia. Previous meta-analyses on this subject have been incomplete in exploring the diverse modalities and treatment parameters of rTMS for post-stroke motor deficits. This study, encompassing the analysis of 49 papers, seeks to assess the effects of rTMS on lower limb motor function in stroke patients at different stages of stroke, aiming to compare the efficacy of various stimulation parameters to formulate a more optimized clinical stimulation protocol.

Drawing from existing literature, the results of this study affirm the substantial efficacy of rTMS in ameliorating post-stroke motor deficits, consistent with the previous findings of Li et al. (68). The analysis reveals that rTMS is most effective in improving FMA-LE in patients with stroke in the subacute phase, while demonstrating significant efficacy in enhancing balance function during the same phase. To enhance walking speed in stroke patients, rTMS proves more effective in those in the acute and subacute stages. However, for patients in the chronic stage, no significant difference was observed between pre and post-treatment. It is crucial to approach this finding with caution, as it may be attributed to the limited number of studies on rTMS intervention in gait for stroke patients in the chronic stage included in this study.

This study systematically investigates the influence of various stimulation parameters on the recovery of lower limb function and provides corresponding optimal stimulation parameters (Table 1). The subgroup analysis reveals that, for patients in the acute stage of stroke, rTMS targeting the unaffected hemisphere proves more effective than stimulating the affected hemisphere in enhancing lower limb function. Conversely, for patients in the subacute stage, stimulating the affected hemisphere demonstrates greater effectiveness. Moreover, research indicates that rTMS intervention in the unaffected hemisphere significantly outperforms rTMS stimulation of either the affected hemisphere or the left DLPFC in improving gait balance and walking speed in stroke patients. This finding’s validity was substantiated in a trial conducted by Shu et al. (69). Their study demonstrated that rTMS targeting the unaffected side substantially impacted the spatiotemporal parameters of gait, influenced joint motion, and affected neurophysiological parameters in stroke patients. Additionally, they observed positive effects on changes in angle and related neurophysiological parameters (MEP latency/CMCT).

**Table 1:** Optimal stimulation parameters.

	Stimulated site	Stimulated brain regions	Stimulation method	rTMS Frequency	Treatment sessions
Stroke/Acute FMA-LE	Unaffected side	M1 OR DLPFC	rTMS	LF-rTMS	20–40
Stroke/Subacute FMA-LE	Affected side	M1	rTMS	HF-rTMS	18
Stroke/chronic FMA-LE	/	/	/	/	/
Balancing Function	Unaffected side	M1	rTMS	LF-rTMS	20–40
Walking Pace	Unaffected side	M1	rTMS	LF-rTMS	20

Previous studies have demonstrated the efficacy of rTMS applied to the M1 region in intervening in neurological disorders such as neuropathic pain, Parkinson’s disease, and post-stroke motor paralysis (70). Consequently, recent research has honed in on rTMS intervention for movement disorders following stroke, particularly targeting the M1 brain region, as evidenced by the results of Lee et al. (71). Their study highlighted the positive impact of HF-rTMS on M1, coupled with treadmill training, on the recovery of lower limb function in chronic stroke patients. Consistent with these findings, our subgroup analysis indicates that patients in the subacute stage of stroke undergoing M1 or DLPFC stimulation exhibit greater improvement in lower limb function compared to those receiving cerebellar stimulation. However, the limited number of studies included in the DLPFC subgroup (only 1) hinders the accumulation of sufficient evidence. Consequently, our study concludes that rTMS stimulation targeting the M1 brain region remains the optimal choice for recovering lower limb function in patients with subacute stroke, extending to the enhancement of balance function in stroke patients. It is noteworthy that rTMS targeting the DLPFC appears to be more effective for lower limb recovery in acute stroke patients.

It is widely recognized that LF-rTMS and cTBS exert an inhibitory effect on stimulated brain areas, whereas HF-rTMS and iTBS have an excitatory effect on stimulated brain areas. According to the currently recognized biphasic competition model in the cerebral hemispheres (72), LF-rTMS or cTBS can inhibit the excitability of the unaffected hemisphere, while HF-rTMS or iTBS can enhance the excitability of the affected hemisphere. All these therapeutic regimens exhibit a certain degree of efficacy. However, no conclusive evidence exists to establish which stimulation modality is most effective in improving post-stroke motor deficits. Therefore, our study conducted a subgroup analysis of different stimulation modalities. The results indicate that both rTMS and TBS significantly contribute to the recovery of lower limb dysfunction in stroke patients. Nevertheless, the present study did not find evidence that iTBS is an effective intervention for improving lower limb function in stroke patients in the subacute phase (*p* = 0.23). The present study is slightly different from the previous meta-analysis by Yuan et al. (73), suggesting that for patients in the acute or subacute phase, conventional rTMS remains the mainstream choice. Additionally, conventional rTMS was more effective than iTBS in improving balance function.

A disagreement exists regarding the optimal stimulation frequency, with some studies suggesting that LF-rTMS can enhance balance, gait ability, and cortical activity in chronic stroke patients (74). Conversely, Wang et al. (75) demonstrated that HF-rTMS was effective in improving walking speed, spatial asymmetry of gait, and lower limb motor function in stroke patients. Therefore, this study scrutinized the efficacy of different rTMS stimulation frequencies, revealing that LF-rTMS was more effective than HF-rTMS in the acute stage of stroke. In contrast, HF-rTMS demonstrated greater efficacy than LF-rTMS in the subacute stage of stroke, with LF-rTMS proving most effective in improving walking balance and walking speed.

Concerning the impact of stimulation sessions on the functional recovery of lower limbs in stroke patients, a previous meta-analysis suggested that the most effective treatment outcomes were observed in patients with stroke duration ≤6 months and treatment duration ≤15 days (76). Moreover, other researchers have indicated that an increase in the number of rTMS sessions leads to a greater improvement in functional balance and postural control after a stroke (77). Our study seeks to investigate the relationship between the recovery of lower limb motor function and stimulation sessions in stroke patients at different stages of stroke. Additionally, our study examines the effect of stimulation sessions on balance and walking speed in stroke patients. The findings of this study suggest that a minimum of 10 stimulations may be associated with more favorable therapeutic outcomes. For patients in the subacute stage, the best therapeutic effects were attained with 18 sessions, and for patients in the acute stage, superior therapeutic effects were observed with stimulation sessions ranging from 20–40. Sessions of 20–40 stimulations also demonstrated superiority in improving balance disorders. Regarding the improvement of walking speed, the study showed that the best results were obtained with 20 stimulation sessions.

However, the current study has certain limitations:

1. Limited subgroup analysis: in this paper, the data were separately analyzed based on lower limb motor function at different stages of stroke, balance function, and gait parameters. However, certain subgroups, such as the impact of rTMS on patients in the chronic stage of stroke, the effects of iTBS/cTBS interventions, stimulation targeting bilateral cerebral hemispheres, and multi-targeted rTMS, were assessed with a limited number of studies. Currently, the evidence from relevant studies is insufficient. Consequently, the reliability of the analyzed results may be somewhat compromised.
2. Analysis of stroke types and causes: this study did not analyze the different types and causes of stroke in the patients. It was not possible to describe the effect of rTMS on strokes triggered by different causes.
3. Heterogeneity and allocation concealment: despite efforts to enhance homogeneity and comparability between studies by analyzing different subgroups, a significant degree of heterogeneity persisted due to variations in interventions within the control groups across the reviewed literature. And the literature included in this paper is partly at risk of bias with unclear allocation concealment.
4. Short-term focus of studies: the majority of current studies only present results after the intervention period or within 1 month after rTMS treatment. The lack of extended follow-up hinders exploration of the long-term effects of rTMS on functional recovery in stroke patients.
5. Limited scope of gait analysis: gait disorders are influenced by multifaceted factors. This study primarily focused on analyzing patients’ FMA, balance, and walking speed. Future research could delve into the effects of rTMS on additional gait parameters, such as step frequency, step length, range of motion of different lower limb joints, and the degree of spasticity in muscle groups.
6. Geographical limitations of the inclusion of literature: in this paper, although the literature search was conducted globally, the literature obtained from the initial search mainly originated from the United States and China. After screening, most of the included literature came from China. This may be related to the large population size of stroke occurrence in China. Therefore, a geographical bias in the analysis is inevitable.

## 5 Conclusion

This systematic evaluation and meta-analysis aimed to explore the impact of rTMS on lower limb function in stroke patients across various stages of the condition, with a specific focus on determining optimal stimulation parameters for this intervention. The results of the study suggest that rTMS proves to be an effective treatment, enhancing FMA-LE scores in stroke patients across all stages. Additionally, it facilitates balance function recovery universally and exhibits advantages in improving walking speed, particularly in acute and subacute stages.

The study conducted an in-depth analysis of stimulation parameters for rTMS intervention in lower limb motor dysfunction following a stroke. The findings revealed that low-frequency stimulation targeting the M1 or DLPFC brain regions of the unaffected hemisphere significantly impacted FMA-LE scores in patients during the acute stage of stroke, with the optimal treatment course identified as 20–40 sessions. For patients in the subacute phase, HF-rTMS directed at the M1 of the affected hemisphere demonstrated superior efficacy in treating lower limb dyskinesia. Optimal results were achieved at 18 sessions.

Furthermore, data analysis indicated that stimulating the M1 brain region of the unaffected hemisphere with LF-rTMS for 20–40 sessions was particularly advantageous in improving the balance disorders of stroke patients. From the perspective of enhancing walking speed, the most effective treatment regimen involved stimulating the M1 brain area of the unaffected hemisphere with LF-rTMS throughout 20 sessions. However, it is crucial to note that the level of evidence for these findings may be influenced by the limited literature analyzed in some subgroups of this study. Therefore, future research should prioritize more high-quality randomized controlled trials (RCTs) to establish the effects of different rTMS parameters on lower limb motor dysfunction in patients with stroke during the chronic phase. Additionally, the validity and reliability of different modalities, diverse targets, and even multi-target approaches of rTMS should be further investigated to offer evidence-based support for the development of clinical rehabilitation programs.

## Data availability statement

The datasets presented in this study can be found in online repositories. The names of the repository/repositories and accession number(s) can be found in the article/Supplementary material.

## Author contributions

SF: Conceptualization, Data curation, Formal analysis, Investigation, Methodology, Project administration, Supervision, Writing – original draft, Writing – review & editing. LY: Conceptualization, Investigation, Methodology, Project administration, Writing – review & editing. JZ: Data curation, Formal analysis, Methodology, Project administration, Resources, Writing – review & editing. YQ: Investigation, Methodology, Project administration, Writing – review & editing. MW: Formal analysis, Resources, Supervision, Visualization, Writing – review & editing. LqY: Data curation, Methodology, Writing – review & editing. TY: Project administration, Resources, Supervision, Writing – review & editing.
