# Supplementary material for: Effects of repetitive transcranial magnetic stimulation on lower extremity motor function and optimal parameters in stroke patients with different stages of stroke: a systematic evaluation and meta-analysis
Source: Front Neurol. 2024 Jul 26;15:1372159. doi: 10.3389/fneur.2024.1372159 (PMC11310066; doi:10.3389/fneur.2024.1372159)
Supplement: Supplementary file 1 [file Data_Sheet_1.docx]

Supplementary Material

# Supplementary Tables

Supplementary Table S1 Pubmed Search Format

| Move | Retrieval Formula |
| --- | --- |
| #1 | Transcranial Magnetic Stimulation［MeSH Terms］ |
| #2 | (“Repetitive Transcranial Magnetic Stimulation”) OR (“theta burst stimulation”) OR (TMS) OR (TBS) |
| #3 | #1 OR #2 |
| #4 | Stroke［MeSH Terms］ |
| #5 | (Strokes) OR (“Cerebrovascular Accident”) OR (“Cerebrovascular Accidents”) OR (CVA) OR (CVAs) OR (“Cerebrovascular Apoplexy”) OR (Apoplexy, Cerebrovascular) OR (“Vascular Accident, Brain”) OR (“Brain Vascular Accident”) OR (“Brain Vascular Accidents”) OR (“Vascular Accidents, Brain”) OR (“Cerebrovascular Stroke”) OR (“Cerebrovascular Strokes”) OR (Stroke, Cerebrovascular) OR (Strokes, Cerebrovascular) OR (Apoplexy) OR (“Cerebral Stroke”) OR (“Cerebral Strokes”) OR (Stroke, Cerebral) OR (Strokes, Cerebral) |
| #6 | #4 OR #5 |
| #7 | Lower extremit［MeSH Terms］ |
| #8 | (“lower limb”) OR (hip or hips) OR (thigh or thighs) OR (knee or knees) OR (leg or legs) OR (ankle or ankles) OR (foot) OR (feet) OR (“lower limbs”) OR (ambula) OR (walk) OR (walking) OR (gait) |
| #9 | #7 OR #8 |
| #10 | #3 AND #6 AND #9 |

Supplementary Table S2 Characteristics of included studies

| STUDY | Design | \| rTMS rTMS \| \| --- \| | | | | Sham rTMS | | | | | rTMS Stimulation Parameters | | | | Outcome measurement | Measure time |
| --- | --- | --- | --- | --- | --- | --- | --- | --- | --- | --- | --- | --- | --- | --- | --- | --- | --- |
|  |  | No. of participants | Mean age (years) | Mean time Post-stroke Stage | Baseline FMA-LE score Subgroup | No. of participants | | Mean age (years) | Mean time Post-stroke Stage | Baseline FMA-LE score Subgroup | rTMS protocol | Sessions | Stimulated site | Stimulated brain regions |  |  |
| Cao 2020 | RCT | 45 | 57.34 | Subacute | 14.27 | | 45 | 57.45 | Subacute | 14.23 | 1Hz ,90%RMT | 20 | Unaffected side | M1 | FMA-LE | Post-intervention |
| Zhu 2023a | RCT | 18 | 60.3 | Acute | 15.4 | 18 | | 61.8 | Acute | 15.6 | 5Hz, 80% RMT, 1000pulses | 40 | Affected side | M1 | FMA-LE, pace | 4w, 8w intervention |
| Zhao 2018 | RCT | 36 | 54 | Acute | 12.72 | 39 | | 54 | Acute | 11.23 | 1Hz ,80%RMT,1000pulses | 20 | Unaffected side | M1 | FMA-LE, BBS | Post-intervention |
| Zeng 2022 | RCT | 40 | 60.22 | Chronic | 17.62 | 40 | | 61.41 | Chronic | 16.34 | 10Hz ,80%RMT | 40 | Affected side | M1 | FMA-LE | 4w, 8w intervention |
| Chen 2018 | RCT | 91 | 56.89 | Acute | 11.25 | 89 | | 57.53 | Acute | 11.47 | 10Hz ,80%RMT | 20 | Left | DLPFC | FMA-LE, BBS | Post-intervention |
| Shan 2022 | RCT | 18 | 56.22 | Subacute |  | 18 | | 55.06 | Subacute |  | 1Hz ,90%RMT,600pulses | 18 | Unaffected side | M1 | pace | Post-intervention |
| Dai 2022 | RCT | 30 | 86.9 | Subacute | 16.67 | 30 | | 87 | Subacute | 16.5 | 1Hz ,90%RMT | 20 | Unaffected side | M1 | FMA-LE | Post-intervention |
| Zhai 2023 | RCT | 23 | 56.26 | Subacute | 13.48 | 23 | | 57.13 | Subacute | 13.39 | 1Hz ,80%RMT | 40 | Unaffected side | M1 | FMA-LE, BBS | Post-intervention |
| Ding 2019 | RCT | 24 | 50.2 | Subacute | 9.44 | 24 | | 48.5 | Subacute | 8.26 | 1Hz ,90%RMT | 24 | Unaffected side | M1 | FMA-LE, BBS, 10mMWS | Post-intervention |
| Ding 2022 | RCT | 38 | 56.65 | Subacute |  | 38 | | 56.31 | Subacute |  | 10Hz ,80%RMT,1200pulses | 21 | Affected side | cerebellum | TUG, BBS | Post-intervention |
| Duan 2020 | RCT | 13 | 46.77 | Subacute | 19.31 | 14 | | 47.86 | Subacute | 18.75 | 1Hz ,80%RMT,1600pulses | 28 | Unaffected side | cerebellum | FMA-LE, FMBS, WGS | Post-intervention |
| Huang 2023a | RCT | 41 | 52.64 | Acute |  | 41 | | 51.73 | Acute |  | 1Hz ,110%RMT | 40 | Unaffected side | M1 | 10 mMWS, BBS, step rate | Post-intervention |
| Huang 2019 | RCT | 47 | 64.7 | Subacute | 14.35 | 45 | | 66.65 | Subacute | 17.59 | 1Hz ,900pulses | 20 | Unaffected side | M1 | FMA-LE | Post-intervention |
| Yu 2022 | RCT | 9 | 54.6 | Acute | 24.01 | 9 | | 57.37 | Acute | 20.27 | 5Hz,80%RMT,1200pulses | 10 | Left | DLPFC | FMA-LE, 10MWT, TUGT, BBS | Post-intervention |
| Liao 2021 | RCT | 15 | 51.53 | Subacute | 22.38 | 15 | | 55.4 | Subacute | 25.31 | iTBS, 80% AMT,600pulses | 10 | Affected side | cerebellum | FMA-LE, BBS | Post-intervention1w, 2w |
| Kim 2014 | RCT | 22 | 67.4 | Acute |  | 10 | | 64.8 | Acute |  | 1Hz ,100%RMT,900pulses | 5 | Unaffected side | cerebellum | 10MWT, BBS | Post-intervention, 1M |
| Cha 2017 | RCT | 10 | 53.8 | Subacute |  | 10 | | 55.8 | Subacute |  | 10Hz ,90%RMT, | 40 | Affected side | M1 | 10 mMWS | Post-intervention |
| Gong 2021 | RCT | 15 | 63.4 | Acute | 15.8 | 15 | | 59.66 | Acute | 15 | 1Hz ,1200pulses | 20 | Unaffected side | M1 | FMA-LE | Follow up 4W, 8W |
| Guan 2017 | RCT | 21 | 59.7 | Acute | 24.2 | 21 | | 57.4 | Acute | 25.6 | 5Hz ,120%RMT,1000pulses | 10 | Affected side | M1 | FMA-LE | Post-intervention2D, Follow up 1M, 3M, 6M, 1N |
| Yu 2023 | RCT | 80 | 65.14 | Subacute | 16.09 | 80 | | 64.23 | Subacute | 15.68 | 1Hz ,90%RMT,1000pulses | 56 | Unaffected side | M1 | FMA-LE, BBS | Post-intervention |
| Yan 2023 | RCT | 88 | 67.82 | Subacute | 16.82 | 88 | | 69.11 | Subacute | 16.97 | 1Hz,70%RMT,50pulses | 30 | Unaffected side | M1 | FMA-LE, BBS, Pace | Post-intervention |
| Jiang 2023 | RCT | 52 | 64.31 | Acute | 13.12 | 52 | | 63.87 | Acute | 13.62 | 10Hz ,90%RMT | 98 | Affected side | DLPFC | FMA-LE | Post-intervention |
| Li 2022 | RCT | 51 | 60.71 | Subacute |  | 52 | | 60.28 | Subacute |  | 0.5Hz ,90%RMT | 20 | Unaffected side | M1 | BBS, Pace | Post-intervention |
| Liang 2023 | RCT | 64 | 55.9 | Acute | 12.63 | 64 | | 57.8 | Acute | 13.09 | 1Hz ,90%RMT,600pulses | 24 | Unaffected side | M1 | FMA-LE, BBS | Post-intervention |
| Xue 2023 | RCT | 25 | 53.72 | Acute | 8.44 | 24 | | 53.13 | Acute | 8.92 | 5Hz/1Hz ,75%RMT,600pulses | 20 | Bilateral | M1 | FMA-LE, BBS, Ankle AROM | Post-intervention |
| Wang 2019 | RCT | 40 | 57.45 | Subacute |  | 40 | | 58.27 | Subacute |  | 1Hz ,110%RMT,300pulses | 40 | Unaffected side | M1 | pace | Post-intervention |
| Tao 2022 | RCT | 20 | 57.5 | Subacute | 20.45 | 20 | | 58.2 | Subacute | 20.1 | 1Hz ,80%RMT,600pulses | 20 | Unaffected side | M1 | FMA-LE, BBS | Post-intervention |
| Ma 2023 | RCT | 20 | 56.75 | Subacute | 14.35 | 20 | | 56.1 | Subacute | 14.2 | 10Hz ,80%RMT,1200pulses | 20 | Affected side | M1 | FMA-LE, step speed, TUGT (rise and walk test) | Post-intervention |
| Tian 2020 | RCT | 22 | 59.18 | Subacute |  | 22 | | 57.09 | Subacute |  | 10Hz ,80%RMT,1200pulses | 24 | Affected side | M1 Lower extremities | BBS, step speed, Functional Ambulation Scale FAC | Post-intervention |
| Mao 2021 | RCT | 41 | 59.12 | Acute | 22.14 | 41 | | 59.33 | Acute | 22.21 | 10Hz,110%RMT,1200pulses | 18 | Affected side | cerebellum | FMA-LE, BBS | Post-intervention |
| Li 2016 | RCT | 30 | 56.7 | Subacute | 21.6 | 30 | | 58 | Subacute | 20.8 | 1Hz ,90%RMT | 20 | Unaffected side | M1 | FMA-LE, 10 mMWS | Post-intervention, Follow up 2W |
| Yang 2016 | RCT | 14 | 56.4 | Subacute | 13.2 | 14 | | 57.5 | Subacute | 14.8 | 10Hz,90%RMT,2000pulses | 24 | Affected side | M1 | FMA-LE, Pace | Post-intervention |
| Yang 2015 | RCT | 60 | 58.7 | Acute | 11.9 | 60 | | 59.2 | Acute | 12 | 2Hz ,90%RMT | 40 | Affected side | M1 | FMA-LE, FAC (Functional Classification of Walking), Pace | Post-intervention, Follow up 4W |
| Wang 2022 | RCT | 21 | 52.62 | Chronic | 18.86 | 21 | | 54.62 | Chronic | 19.1 | iTBS, 80% RMT,600pulses | 20 | Affected side | cerebellum | FMA-LE, BBS | Post-intervention |
| Shi 2021 | RCT | 20 | 59.3 | Subacute | 14.95 | 20 | | 60.2 | Subacute | 15.3 | 1Hz ,80%RMT,600pulses | 40 | Unaffected side | M1 | FMA-LE, BBS | Post-intervention |
| Yang 2020 | RCT | 34 | 61.98 | Subacute | 17.42 | 34 | | 61.52 | Subacute | 17.83 | 10Hz ,80%RMT | 48 | Affected side | DLPFC | FMA-LE | Post-intervention |
| Liu 2023 | RCT | 30 | 57.73 | Subacute |  | 30 | | 56.36 | Subacute |  | 5Hz,80%RMT,1200pulses | 14 | Left | DLPFC | 10MWT, TUGT, BBS | Post-intervention |
| Ni 2021 | RCT | 48 | 68.87 | Subacute | 19.06 | 48 | | 65.34 | Subacute | 18.59 | 1Hz ,100%RMT | 72 | Unaffected side | M1 | FMA-LE, BBS, Pace | Post-intervention |
| Tang 2022 | RCT | 40 | 55.92 | Acute | 14.85 | 40 | | 58.92 | Acute | 14.37 |  | 20 | Bilateral | M1 | FMA-LE, FAC, BBS | Post-intervention |
| Wang 2023a | RCT | 18 | 56.72 | Subacute | 13.5 | 18 | | 53.44 | Subacute | 14.11 | 10Hz ,80%RMT,1200pulses | 18 | Affected side |  | FMA-LE, BBS | Post-intervention |
| Wang 2023a1 | RCT | 18 | 54.83 | Subacute | 13.78 | 18 | | 53.44 | Subacute | 14.11 | iTBS, 70% RMT, 1200pulses | 18 | Affected side |  | FMA-LE, BBS | Post-intervention |
| Wang 2021 | RCT | 29 | 50.96 | Subacute | 19.23 | 31 | | 51.36 | Subacute | 19.78 | 1Hz ,90%RMT | 24 | Unaffected side | M1 | FMA-LE | Post-intervention |
| Qiao 2023 | RCT | 61 | 54.21 | Subacute | 17.14 | 61 | | 55.36 | Subacute | 16.49 | 1Hz ,80%RMT | 20 | Unaffected side | M1 | FMA-LE | Post-intervention |
| Zhu 2023b | RCT | 34 | 64.6 | Subacute | 14.15 | 20 | | 64.7 | Subacute | 14.11 | 1Hz ,90%RMT,1200pulses | 15 | Unaffected side | M1 | FMA-LE, BBS | Post-intervention |
| Wang 2023b | RCT | 80 | 63.85 | Acute | 11.73 | 80 | | 64.1 | Acute | 11.69 | 10Hz | 18 | Unffected side | M1 | FMA-LE, BBS | Post-intervention |
| Wang 2023b1 | RCT | 80 | 63.92 | Acute | 11.6 | 80 | | 64.1 | Acute | 11.69 | 0.5Hz | 18 | Affected side | M1 | FMA-LE, BBS | Post-intervention |
| Wang 2012 | RCT | 12 | 64.9 | Chronic | 17.58 | 12 | | 62.98 | Chronic | 18.17 | 1Hz ,90%RMT,600pulses | 10 | Unaffected side | M1 Lower extremities | FMA-LE, Pace | Post-intervention |
| Sharma 2020 | RCT | 47 | 54.85 | Subacute | 27.61 | 49 | | 52.89 | Subacute | 28.53 | 1Hz ,110%RMT,750pulses | 10 | Unaffected side | M1 | FMA-LE | Post-intervention |
| Liu 2024 | RCT | 20 | 59.7 | Subacute | 17.5 | 20 | | 60.7 | Subacute | 17 | 3HZ,80%RMT,1800pulses | 10 | Affected side | M1 | FMA-LE BBS | Post-intervention |
| Zhu 2024 | RCT | 18 | 58.67 | Subacute | 20.57 | 18 | | 62.33 | Subacute | 23.1 | iTBS ,80%AMT,1200pulses | 10 | Affected side | Cerebellar | FMA-LE BBS Pace | Post-intervention |
| Wang 2024 | RCT | 27 | 60.95 | Acute | 17.78 | 25 | | 61.76 | Acute | 18.01 | 1Hz, 90%RMT,600pulses | 30 | Unaffected side | M1 | FMA-LE BBS Pace | Post-intervention |

# Supplementary Table S3 The Mean and SD of all Experimental and Control Groups

# FMA-LE

| Author | Year | Experimental Mean | Experimental SD | Total | Control Mean | Control SD | Total | Stage |
| --- | --- | --- | --- | --- | --- | --- | --- | --- |
| Zhu | 2023a | 20.8 | 2.4 | 18 | 20.1 | 2.8 | 18 | Acute |
| Chen | 2018 | 17.83 | 5.06 | 91 | 13.15 | 3.78 | 89 | Acute |
| Zhai | 2023 | 23.83 | 2.64 | 23 | 19.39 | 2.87 | 23 | Acute |
| Gong | 2021 | 18.26 | 4.87 | 15 | 16.8 | 5.6 | 15 | Acute |
| Jiang | 2023 | 22.71 | 3.64 | 52 | 19.96 | 3.26 | 52 | Acute |
| Liang | 2023 | 26.79 | 6.08 | 64 | 19.84 | 5.93 | 64 | Acute |
| Xue | 2023 | 20.72 | 3.9 | 25 | 14.5 | 2.74 | 24 | Acute |
| Mao | 2021 | 27.26 | 2.54 | 41 | 24.68 | 2.66 | 41 | Acute |
| Yang | 2015 | 25.7 | 3.89 | 60 | 18.5 | 4.04 | 60 | Acute |
| Tang | 2022 | 23.47 | 4.74 | 40 | 20.45 | 6.13 | 40 | Acute |
| Wang | 2023b | 16.02 | 3.38 | 80 | 14.93 | 3.44 | 80 | Acute |
| Wang | 2023b1 | 18.71 | 4.78 | 80 | 14.93 | 3.44 | 80 | Acute |
| Zhao | 2018 | 17.92 | 9.49 | 36 | 14.21 | 8.08 | 39 | Acute |
| Yu | 2022 | 31.95 | 10.28 | 9 | 29.82 | 9.25 | 9 | Acute |
| Guan | 2017 | 29.9 | 2.4 | 21 | 29.2 | 1.8 | 21 | Acute |
| Wang | 2024 | 30.64 | 4.17 | 27 | 25.53 | 5.95 | 25 | Acute |
| Cao | 2020 | 24.23 | 4.56 | 45 | 18.78 | 4.23 | 45 | Subacute |
| Shi | 2021 | 22.05 | 2.93 | 20 | 19.15 | 3.44 | 20 | Subacute |
| Yang | 2020 | 25.87 | 6.33 | 34 | 20.38 | 5.8 | 34 | Subacute |
| Wang | 2021 | 31.18 | 2.4 | 29 | 24.09 | 3.78 | 31 | Subacute |
| Zhu | 2023 | 28.76 | 5.67 | 34 | 23.66 | 4.8 | 20 | Subacute |
| Ni | 2021 | 27.05 | 3.69 | 48 | 24.85 | 3.35 | 48 | Subacute |
| Sharma | 2020 | 30.29 | 4.73 | 47 | 29.73 | 6.1 | 49 | Subacute |
| Li | 2016 | 28.2 | 2.8 | 30 | 25.4 | 3.3 | 30 | Subacute |
| Wang | 2023a | 25.11 | 5.99 | 18 | 18.83 | 5.71 | 18 | Subacute |
| Wang | 2023a1 | 25.94 | 6.19 | 18 | 18.83 | 5.71 | 18 | Subacute |
| Yang | 2016 | 28.6 | 3.95 | 14 | 22.9 | 4.1 | 14 | Subacute |
| Ding | 2019 | 26.47 | 3.59 | 24 | 14.65 | 2.38 | 24 | Subacute |
| Qiao | 2023 | 27.83 | 4.06 | 61 | 24.53 | 3.87 | 61 | Subacute |
| Duan | 2020 | 26.18 | 1.24 | 13 | 24.73 | 1.42 | 14 | Subacute |
| Dai | 2022 | 26.27 | 4.42 | 30 | 22.6 | 7.01 | 30 | Subacute |
| Ma | 2023 | 26.95 | 2.86 | 20 | 21.45 | 2.35 | 20 | Subacute |
| Tao | 2022 | 23.85 | 2.5 | 20 | 22 | 2.88 | 20 | Subacute |
| Yan | 2023 | 29.18 | 4.81 | 88 | 24.13 | 4.52 | 88 | Subacute |
| Yu | 2023 | 28.23 | 6.74 | 80 | 22.45 | 4.36 | 80 | Subacute |
| Huang | 2019 | 20.77 | 8.4 | 47 | 17.59 | 8.04 | 45 | Subacute |
| Liu | 2024 | 25.6 | 3.9 | 20 | 22.4 | 4.2 | 20 | Subacute |
| Zhu | 2024 | 25.11 | 5.86 | 18 | 24.63 | 4.59 | 18 | Subacute |
| Zeng | 2022 | 27.8 | 5.3 | 40 | 22.79 | 6.3 | 40 | Chronic |
| Wang | 2022 | 29.76 | 2.19 | 21 | 26.95 | 2.91 | 21 | Chronic |
| Wang | 2012 | 22.92 | 4.66 | 12 | 21.92 | 7.12 | 12 | Chronic |

# (B) Balance

| Author | Year | Experimental Mean | Experimental SD | Total | Control Mean | Control SD | Total |
| --- | --- | --- | --- | --- | --- | --- | --- |
| Zhao | 2018 | 15.13 | 12.85 | 36 | 8.73 | 10.11 | 39 |
| Chen | 2018 | 53.79 | 17.68 | 91 | 45.82 | 14.75 | 89 |
| Zhai | 2023 | 36.17 | 3.1 | 23 | 31.65 | 4.31 | 23 |
| Ding | 2019 | 46.71 | 2.68 | 24 | 34.05 | 3.21 | 24 |
| Ding | 2022 | 45.81 | 5.07 | 38 | 40.49 | 5.44 | 38 |
| Duan | 2020 | 8.38 | 1.14 | 13 | 6.73 | 1.03 | 14 |
| Huang | 2023a | 35.12 | 5.17 | 41 | 27.69 | 4.69 | 41 |
| Yu | 2022 | 52.37 | 13.48 | 9 | 46.79 | 12.51 | 9 |
| Liao | 2021 | 49.92 | 3.53 | 15 | 46.75 | 7.44 | 15 |
| Kim | 2014 | 30.8 | 15.8 | 22 | 32.6 | 16.5 | 10 |
| Yu | 2023 | 46.91 | 7.27 | 80 | 35.71 | 5.56 | 80 |
| Yan | 2023 | 48.38 | 6.32 | 88 | 42.1 | 6.04 | 88 |
| Li | 2022 | 47.29 | 4.95 | 51 | 39.64 | 4.65 | 52 |
| Liang | 2023 | 11.56 | 5.91 | 64 | 7.3 | 3.29 | 64 |
| Xue | 2023 | 29.72 | 6.41 | 25 | 20.29 | 6.16 | 24 |
| Tao | 2022 | 33.55 | 5.87 | 20 | 30.4 | 3.97 | 20 |
| Tian | 2020 | 23.73 | 8.42 | 22 | 18.5 | 7.39 | 22 |
| Mao | 2021 | 46.57 | 5.21 | 41 | 39.11 | 4.86 | 41 |
| Wang | 2022 | 52.14 | 2.15 | 21 | 49.86 | 2.71 | 21 |
| Shi | 2021 | 39.15 | 2.68 | 20 | 35.65 | 3.69 | 20 |
| Liu | 2023 | 44.25 | 4.14 | 30 | 41.31 | 4.42 | 30 |
| Ni | 2021 | 41.15 | 4.97 | 48 | 36.08 | 4.58 | 48 |
| Tang | 2022 | 23.57 | 8.64 | 40 | 31.67 | 9.98 | 40 |
| Wang | 2023a | 35.89 | 11.63 | 18 | 22.56 | 12.01 | 18 |
| Wang | 2023a1 | 35.89 | 11.39 | 18 | 22.56 | 12.01 | 18 |
| Zhu | 2023b | 36.75 | 5.65 | 34 | 30.32 | 3.38 | 20 |
| Wang | 2023b | 24.43 | 5.87 | 80 | 19.18 | 4.8 | 80 |
| Wang | 2023b1 | 24.72 | 5.16 | 80 | 19.18 | 4.8 | 80 |
| Wang | 2024 | 42.04 | 6.31 | 27 | 32.87 | 5.67 | 25 |
| Zhu | 2024 | 37.47 | 12.08 | 18 | 34.58 | 8.34 | 18 |
| Liu | 2024 | 36.7 | 8.9 | 20 | 31 | 5.5 | 20 |

# (C) Walking speed

| Author | Year | Experimental Mean | Experimental SD | Total | Control Mean | Control SD | Total |
| --- | --- | --- | --- | --- | --- | --- | --- |
| Zhu | 2023a | 34.4 | 5.6 | 18 | 33.9 | 4.8 | 18 |
| Shan | 2022 | 47.94 | 13.44 | 18 | 39.94 | 9.52 | 18 |
| Ding | 2019 | 58.63 | 6.55 | 24 | 46.05 | 6.37 | 24 |
| Huang | 2023a | 51.04 | 9.14 | 41 | 40.81 | 7.23 | 41 |
| Yan | 2023 | 153 | 43 | 88 | 124 | 40 | 88 |
| Li | 2022 | 77.92 | 14.31 | 51 | 69.46 | 15.47 | 52 |
| Wang | 2019 | 60.28 | 8.2 | 40 | 55.5 | 9 | 40 |
| Tian | 2020 | 110 | 32 | 22 | 122 | 40 | 22 |
| Yang | 2016 | 78.52 | 17.45 | 14 | 69.1 | 15.23 | 14 |
| Yang | 2015 | 44 | 13 | 60 | 33 | 8 | 60 |
| Ni | 2021 | 93 | 30 | 48 | 80 | 25 | 48 |
| Wang | 2012 | 76.32 | 23.94 | 12 | 59.15 | 19.93 | 12 |
| Cha | 2017 | 84 | 32 | 10 | 55 | 0.26 | 10 |
| Ma | 2023 | 26.95 | 2.86 | 20 | 21.45 | 2.35 | 20 |
| Li | 2016 | 56.78 | 20.15 | 30 | 42.46 | 17.91 | 30 |
| Wang | 2024 | 75.03 | 11.36 | 27 | 58.85 | 9.87 | 25 |
| Zhu | 2024 | 62.33 | 35.19 | 18 | 46.39 | 21.42 | 18 |

# Supplementary Figures


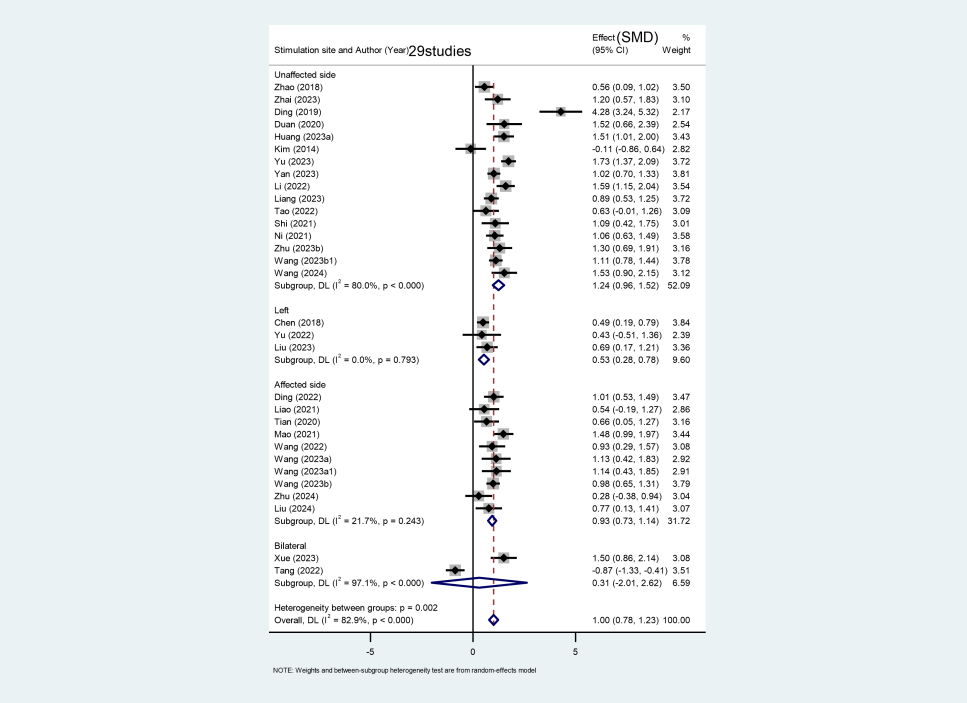

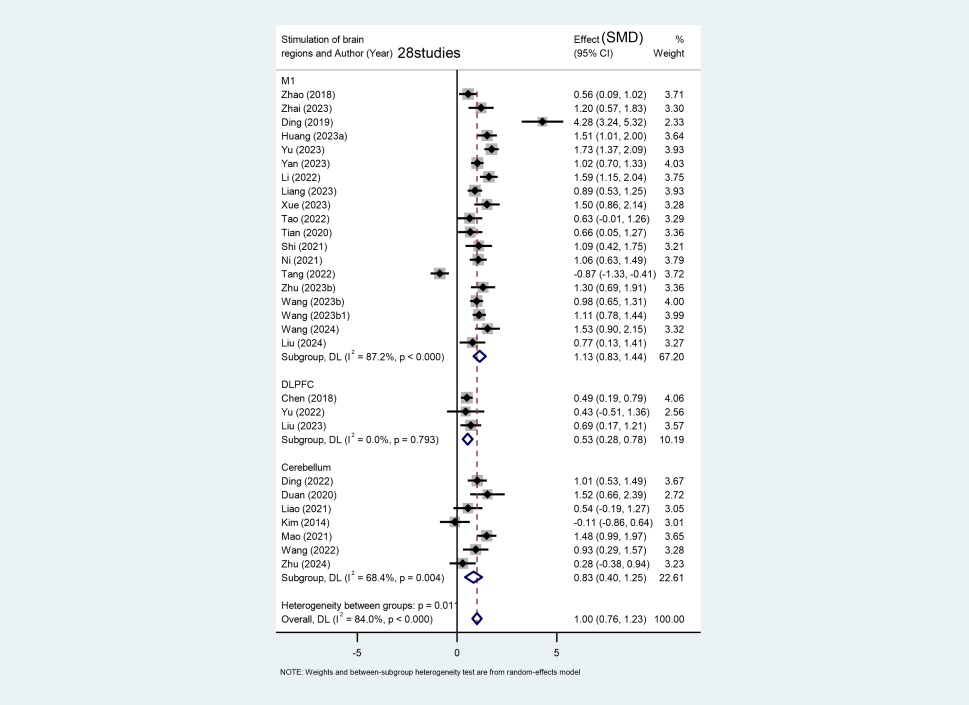


A B


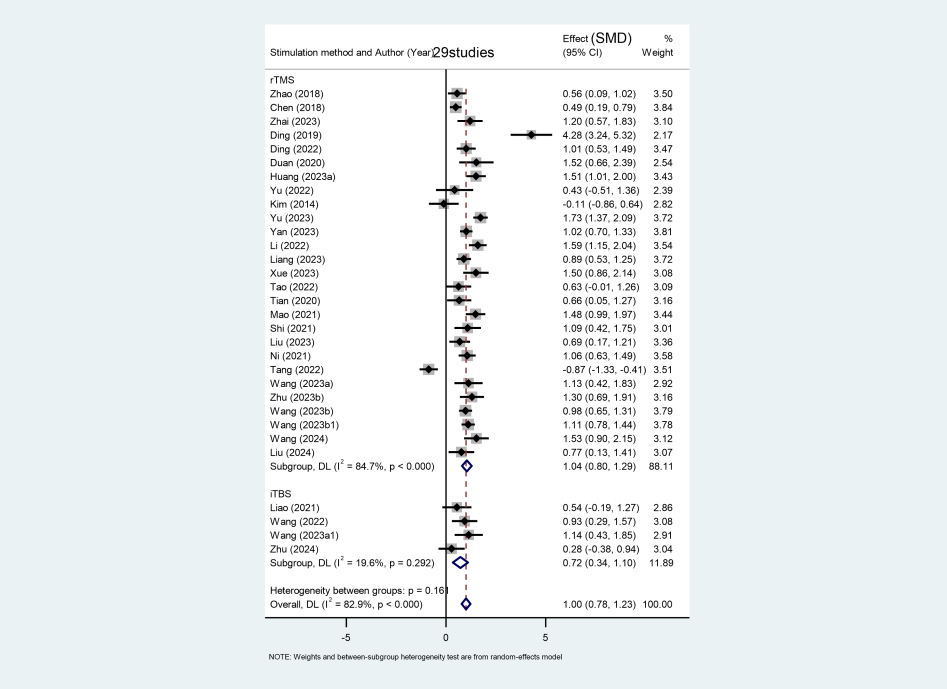

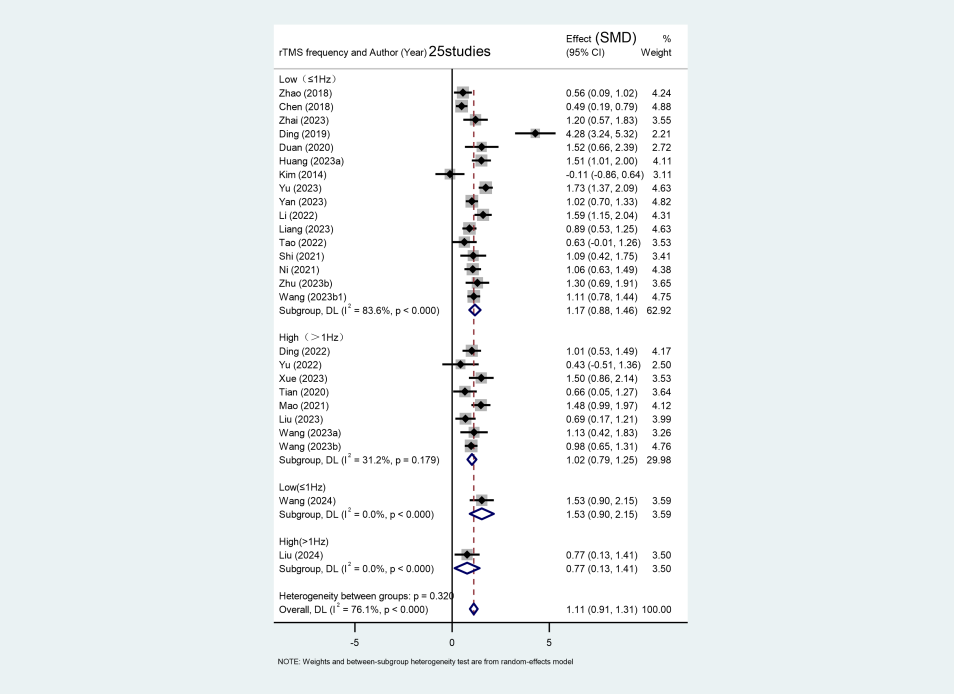


C D


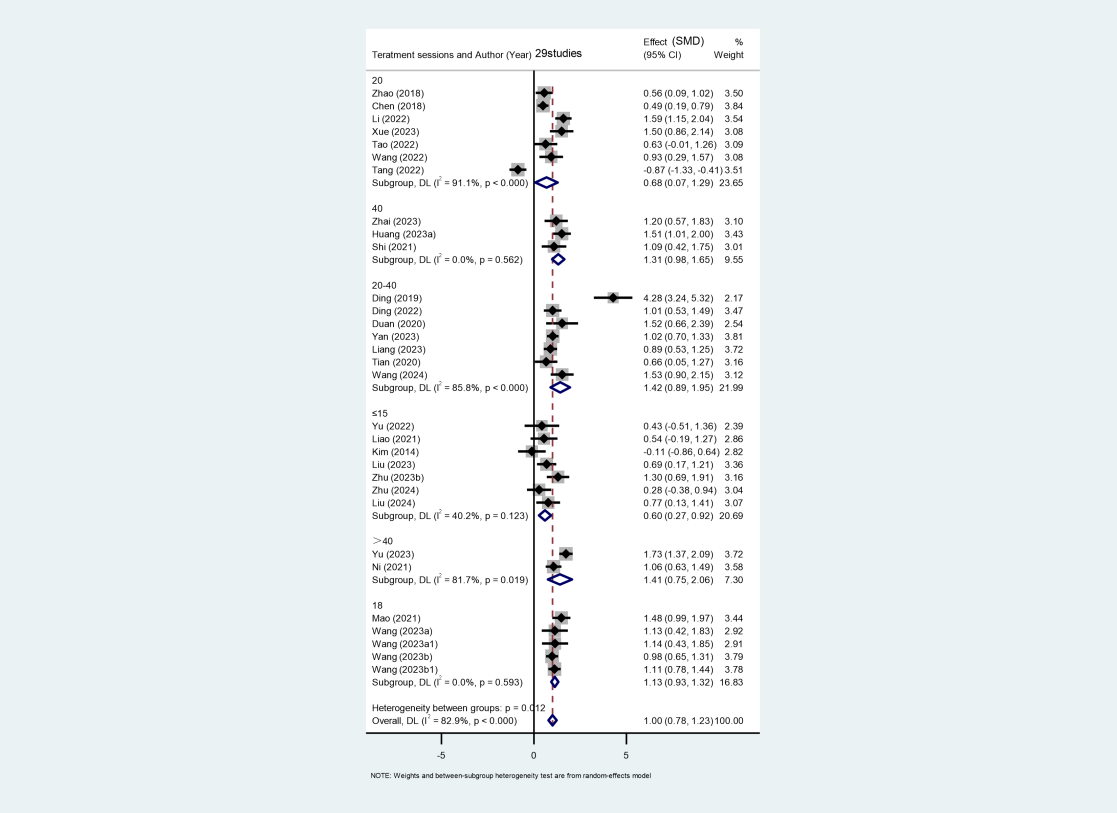


E


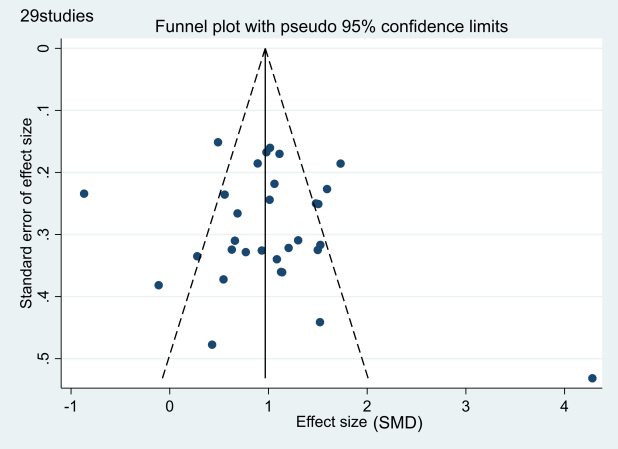

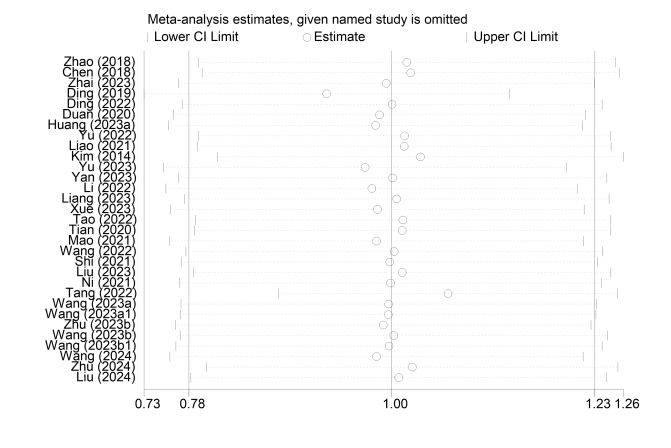


F G

Supplementary Figure 1. (A)Forest plot of balance function in stroke patients disaggregated by stimulate site compared with controls.(B)Forest plot of balance function in stroke patients disaggregated by stimulate of brain regions compared with controls.(C)Forest plot of balance function in stroke patients disaggregated by stimulate method compared with controls.(D)Forest plot of balance function in stroke patients disaggregated by stimulate frequence compared with controls.(E)Forest plot of balance function in stroke patients disaggregated by stimulate sessions compared with controls.(F)Funnel plot of balance function in stroke patients.(G)Results of sensitivity analysis of balance function in stroke patients.


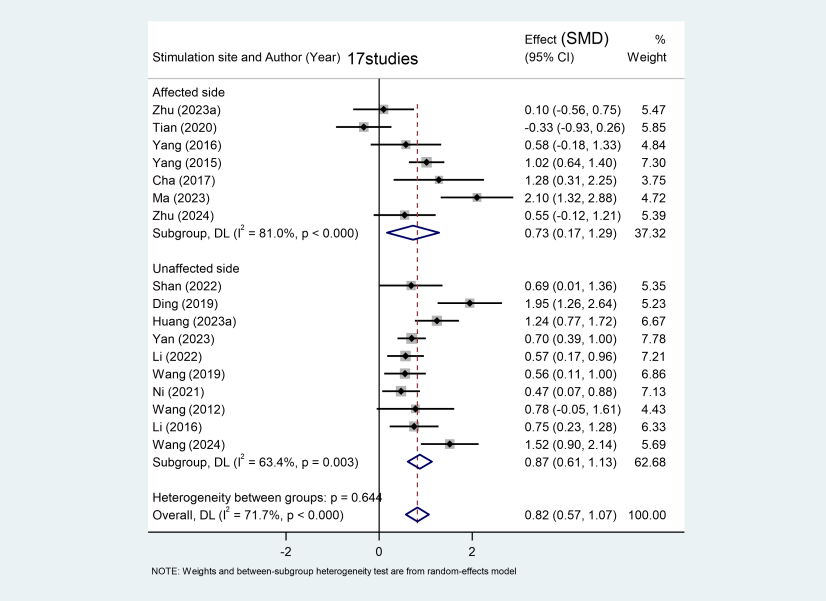


A B


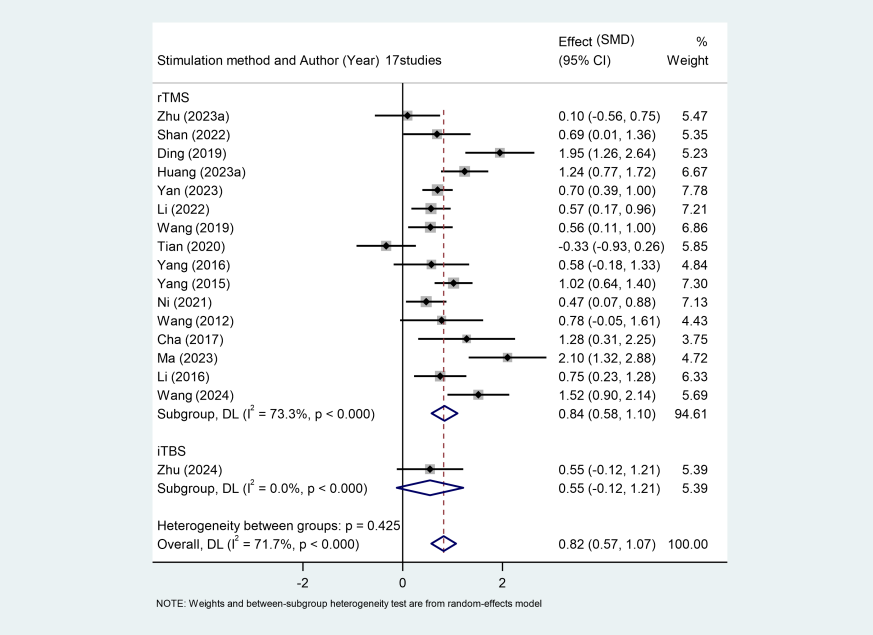


C D


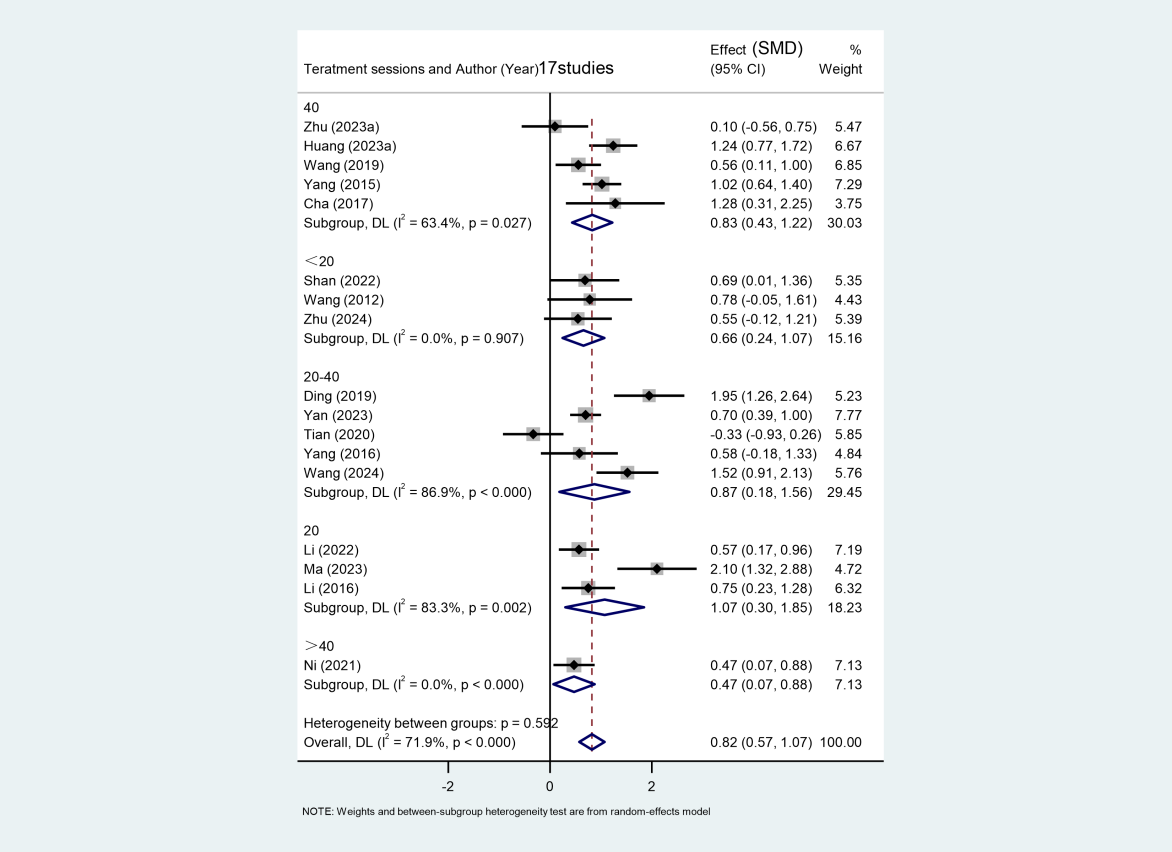


E


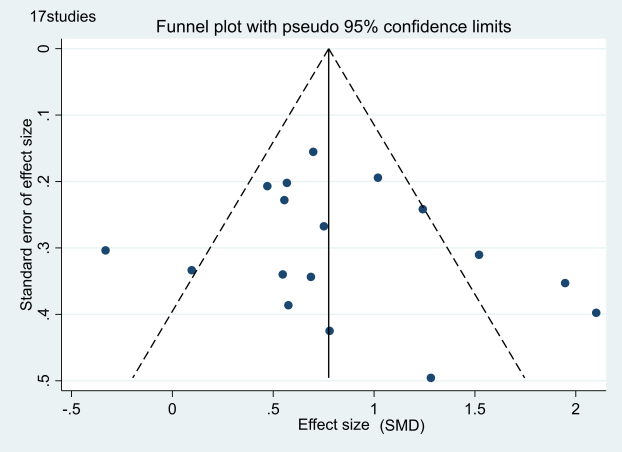

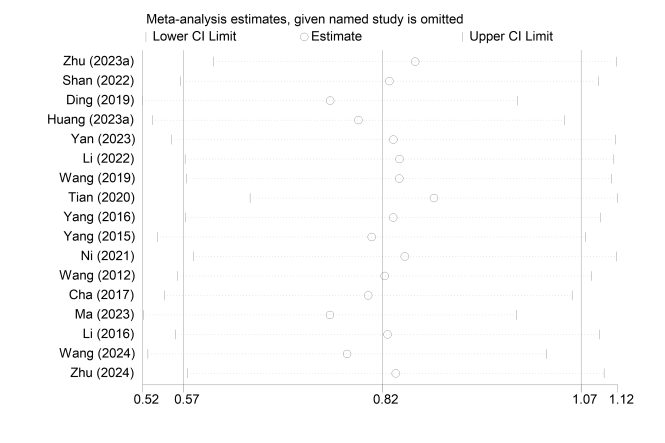


F G

Supplementary Figure 2.(A)Forest plot of walking pace in stroke patients disaggregated by stimulate site compared with controls.(B)Forest plot of walking pace in stroke patients disaggregated by stimulate of brain regions compared with controls.(C)Forest plot of walking pace in stroke patients disaggregated by stimulate method compared with controls.(D)Forest plot of walking pace in stroke patients disaggregated by stimulate frequence compared with controls.(E)Forest plot of walking pace in stroke patients disaggregated by stimulate sessions compared with controls.(F)Funnel plot of walking pace in stroke patients.(G)Results of sensitivity analysis of walking pace in stroke patients.
